# MiR-199a-3p enhances cisplatin sensitivity of cholangiocarcinoma cells by inhibiting mTOR signaling pathway and expression of MDR1

**DOI:** 10.18632/oncotarget.16834

**Published:** 2017-04-04

**Authors:** Qiang Li, Xuefeng Xia, Jie Ji, Jianghui Ma, Liang Tao, Linjun Mo, Wei Chen

**Affiliations:** ^1^ Department of General Surgery, The Afflicted Drum Tower Hospital of Nanjing University Medical School, Nanjing, China; ^2^ Nangjing Medical University, Nangjing, China; ^3^ School of Surgery, The University of Western Australia, and Western Australia Liver and Kidney Surgical Transplant Service, Sir Charles Gairdner Hospital, Perth, Western Australia, Australia; ^4^ Institute of Molecular Engineering, University of Chicago, Chicago, Illinois, USA

**Keywords:** MiR-199a-3p, cholangiocarcinoma, mTOR, MDR1, chemosensitivity

## Abstract

Several studies have reported reduced miRNA-199a-3p (miR-199a-3p) in different human malignancies, however, little is known about miR-199a-3p in cholangiocarcinoma cells. In this study, we demonstrate the essential role and mechanism of miR-199a-3p in regulating cisplatin sensitivity in cholangiocarcinoma cell lines. Using a CCK-8 cell counting assay we found that expression of miR-199a-3p was positively correlated with cisplatin sensitivity in cholangiocarcinoma cell lines. MiR-199a-3p overexpression could decrease the proliferation rate and increase apoptosis of cholangiocarcinoma cells in the presence of cisplatin, while miR-199a-3p inhibition had the opposite effect. Further study demonstrated that mTOR was the target gene of miR-199a-3p, and that miR-199a-3p mimics could inhibit expression of mTOR, which consequently reduced the phosphorylation of its downstream proteins 4EBP1 and p70s6k. Rescue experiments proved that miR-199a-3p could increase the cisplatin sensitivity of cholangiocarcinoma cell lines by regulating mTOR expression. Moreover, we also found that miR-199a-3p overexpression could reduce cisplatin induced MDR1 expression by decreasing the synthesis and increasing the degradation of MDR1, thus enhancing the effectiveness of cisplatin in cholangiocarcinoma. In conclusion, miR-199a-3p could increase cisplatin sensitivity of cholangiocarcinoma cell lines by inhibiting the activity of the mTOR signaling pathway and decreasing the expression of MDR1.

## INTRODUCTION

As a bile duct epithelium cancer, cholangiocarcinoma is the second most common primary hepatic malignancy after hepatocellular carcinoma, and accounts for 10–20% of primary liver cancers [1, 2]. Even amongst those able to undergo radical resection, the recurrence risk is high and the 5-year overall survival is only 30% [3]. One of the first-line chemotherapeutic agents, cisplatin, has been universally found to benefit individuals with advanced, unresectable or metastatic gallbladder cancer [4, 5]. The current standard first-line treatment for unresectable CCA is combination treatment with gemcitabine and cisplatin, however, drug resistance is common and the prognosis of patients is universally poor [6]. Future studies which focus on how to eliminate the incidence of drug resistance are essential.

Mammalian target of rapamycin (mTOR), an atypical serine/threonine kinase, is activated in numerous cancers, regulating cell proliferation, growth, differentiation, migration, and survival [7]. Recent studies have revealed that inhibition of mTOR signaling using rapamycin can enhance sensitivity of cancer cells to cisplatin and doxorubicin [8, 9]. Our previous studies also revealed that inhibition of mTOR suppresses human gallbladder carcinoma cell proliferation and enhances the cytotoxicity of 5-fluorouracil (5-FU) by regulating MDR1 expression [10]. However, little is known about the role of mTOR in cholangiocarcinoma.

Evidence collected to date shows that microRNAs can regulate the mTOR signaling pathway and affect cancer cell sensitivity to chemotherapy drugs [11]. MiR-199a-3p has been shown to have significantly downregulated expression in several cancers [12–17]. Several previous studies indicated that miR-199a-3p may target mTOR and affect cell proliferation, and that drug resistance is modulated through inhibition of mTOR [18–22]. However, in cholangiocarcinoma, the role of miR-199a-3p and its relationship with mTOR are unknown.

In this study, we found miR-199a-3p was upstream of the mTOR signaling pathway. Further, we demonstrate that miR-199a-3p could increase the cisplatin sensitivity of cholangiocarcinoma cell lines by inhibiting the activity of the mTOR signaling pathway, decreasing the synthesis of MDR1, and increasing the degradation of MDR1.

## RESULTS

### Expression of miR-199a-3p was negatively correlated with cisplatin sensitivity in cholangiocarcinoma cells

**Figure 1:**
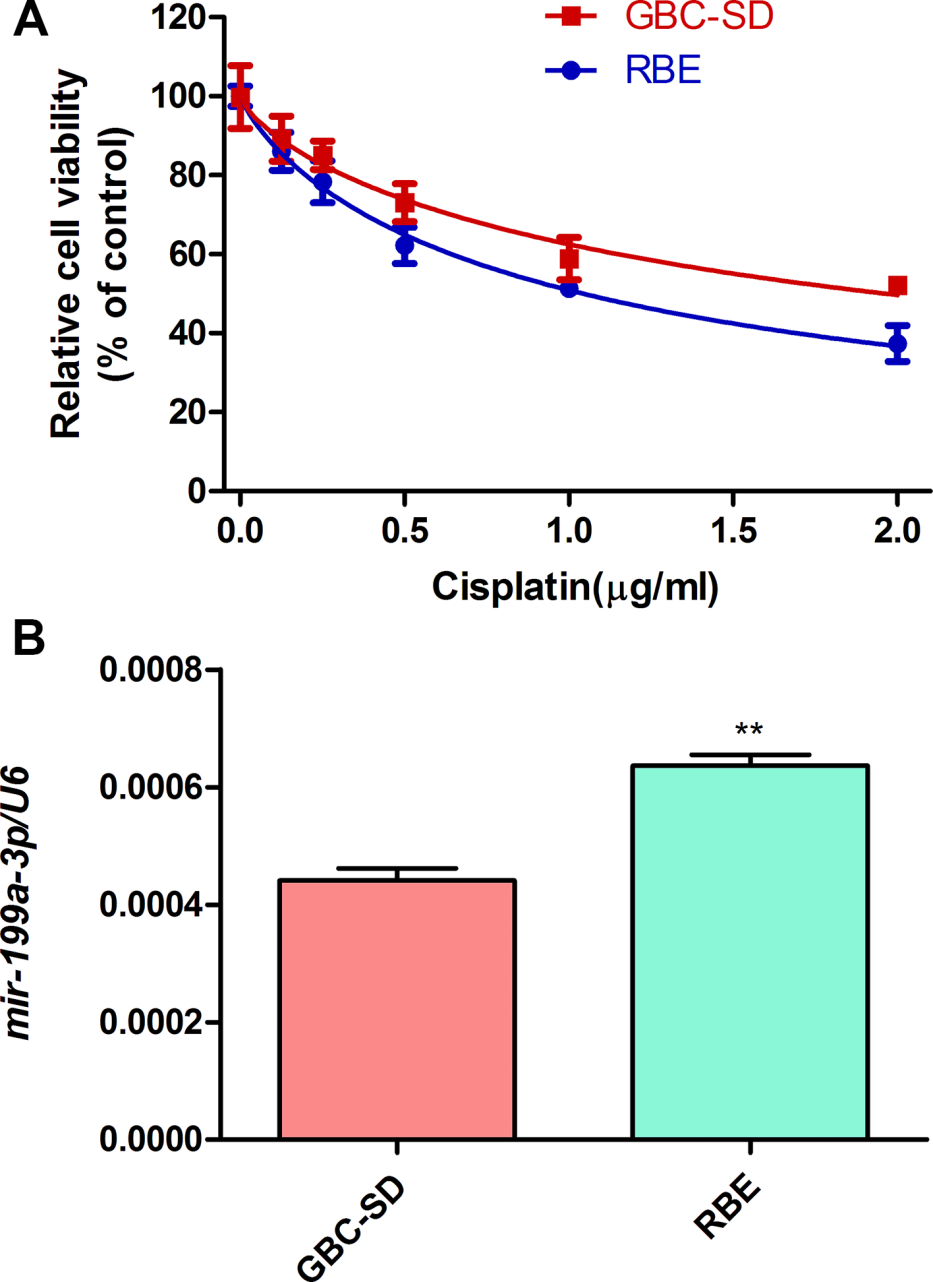
Cell viability under cisplatin and miR-199a-3p expression of cholangiocarcinoma cells (**A**) Cell viability of GBC-SD and RBE cell lines under different concentration of cisplatin by CCK-8 assay. (**B**) Expression of miR-199a-3p in GBC-SD and RBE cell lines examined by qPCR. U6 was used as the internal reference. **P < 0.01 vs. GBC-SD.

To investigate the function of miR-199a-3p in cisplatin sensitivity of cholangiocarcinoma cell lines, we used a CCK-8 assay to study the cell viability of cholangiocarcinoma cells (GBC-SD and RBE) under different concentrations of cisplatin. We found the viability of those cell lines declined depending on the cisplatin concentration (0 μg/ml, 0.5 μg/ml, 1.0 μg/ml, 1.5 μg/ml, 2.0 μg/ml) (Figure 1A). RBE cells were more sensitive to cisplatin than GBC-SD cells (RBE IC_50_: 1.044 μg/ml; GBC-SD IC_50_: 1.966 μg/ml). Interestingly, the RBE cell line, which had higher expression of miR-199a-3p (Figure 1B, **P < 0.01 vs. GSC-SD), was more sensitive to cisplatin. Thus, high expression of miR-199a-3p may lead to greater sensitivity to cisplatin treatment.

### MiR-199a-3p could regulate the cisplatin sensitivity of cholangiocarcinoma cell lines

**Figure 2:**
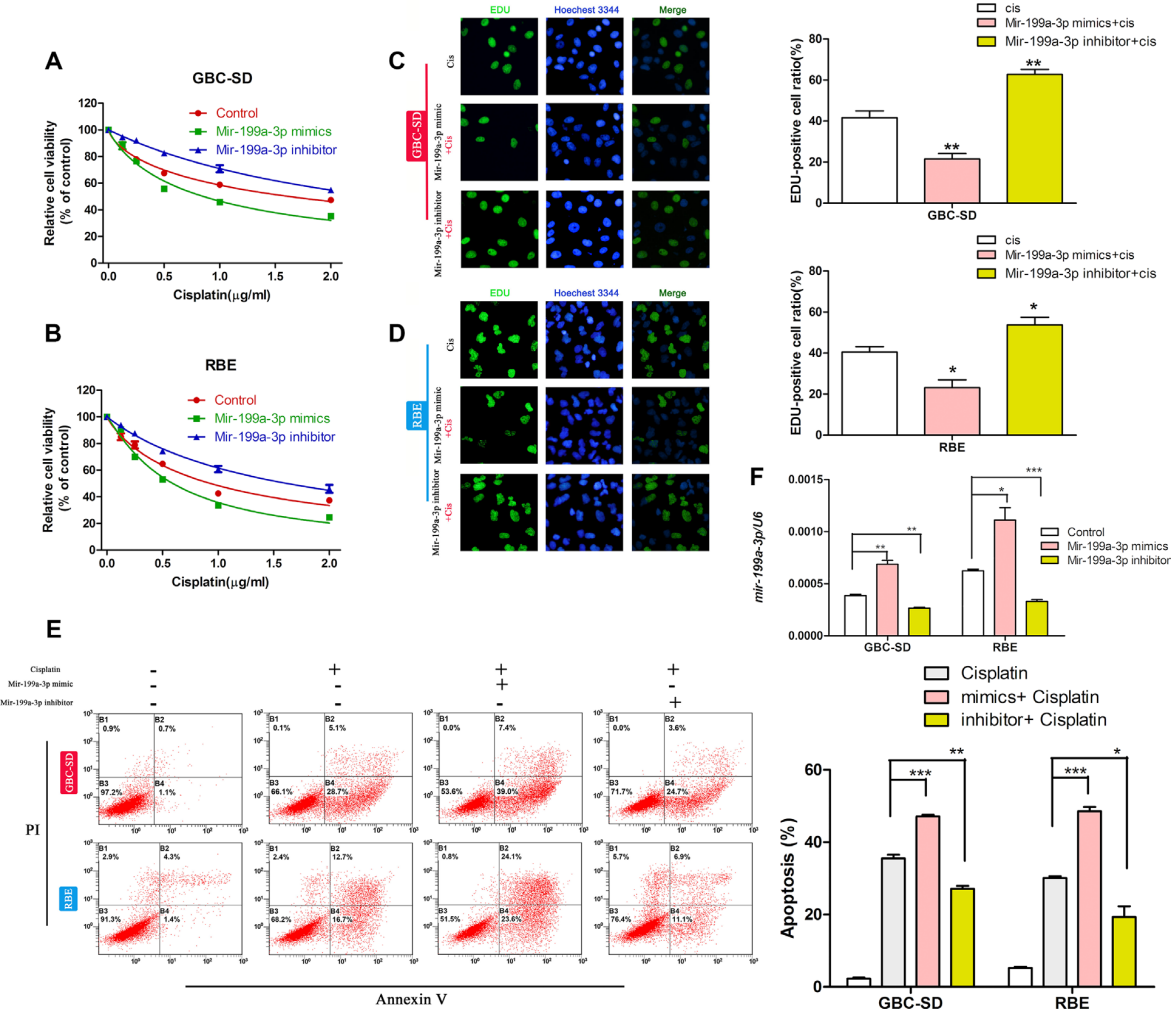
MiR-199a-3p enhanced cisplatin sensitivity of cholangiocarcinoma cells (**A**–**B**) Cell viability under different concentrations of cisplatin of GBC-SD and RBE cell lines treated with miR-199a-3p mimics, inhibitor and negative control, examined by CCK-8 assay. (**C**–**D**) Cell proliferation rate under different concentrations of cisplatin of GBC-SD and RBE cell lines treated with miR-199a-3p mimics, inhibitor and negative control, examined by EdU assay. The number of EdU positive cells was counted. (**E**) Apoptosis incidence under certain concentrations of cisplatin in GBC-SD and RBE cell lines treated with miR-199a-3p mimics, inhibitor and negative control, examined by flow cytometry. The cell numbers in quadrants Q2 and Q4 were defined as apoptotic cells. *P < 0.05, **P < 0.01, ***P < 0.001.

To confirm our discovery, we used miR-199a-3p mimics and miR-199a-3p inhibitors. We first studied the cell viability of cholangiocarcinoma cell lines with different miR-199a-3p expression under different concentrations of cisplatin by CCK-8 assay. We found that miR-199a-3p mimics could improve the toxicity of cisplatin in GBC-SD and RBE cell lines when compared to the negative controls, while the miR-199a-3p inhibitor led to the opposite result (Figure 2A–2B) (*P < 0.05,**P < 0.01, ***P < 0.001). EdU assay revealed that miR-199a-3p mimics could decrease the proliferation rate of cholangiocarcinoma cell lines under treatment with cisplatin when compared to the negative controls, while the miR-199a-3p inhibitor showed the opposite effect (Figure 2C–2D). The results of flow cytometry assays further confirmed that miR-199a-3p could enhance the sensitivity of cholangiocarcinoma cell lines to cisplatin (Figure 2E) (*P < 0.05 **P < 0.01, ***P < 0.001).

### mTOR is the target gene of miR-199a-3p

**Figure 3:**
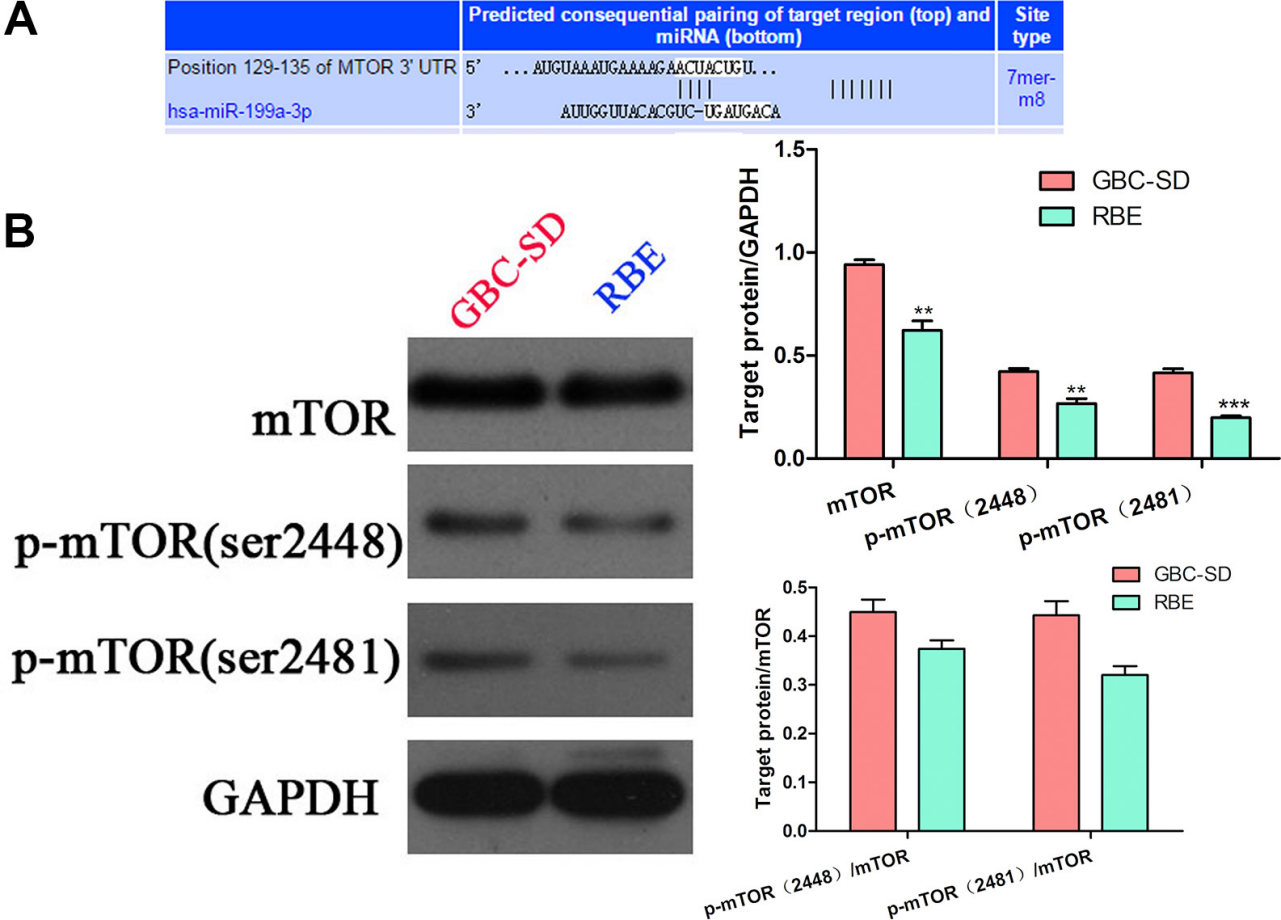
mTOR expression in cholangiocarcinoma cells and the TargetScan result (**A**) TargetScan predicted mTOR was the target gene of miR-199a-3p: miR-199a-3p could bind the 129–135 positions of the mTOR 3′-UTR. (**B**) Western blotting detected the expression of mTOR and p-mTOR (sites 2481 and 2448) in GBC-SD and RBE cell lines; the results were quantified using ImageJ software (**P < 0.01, ***P < 0.001).

**Figure 4:**
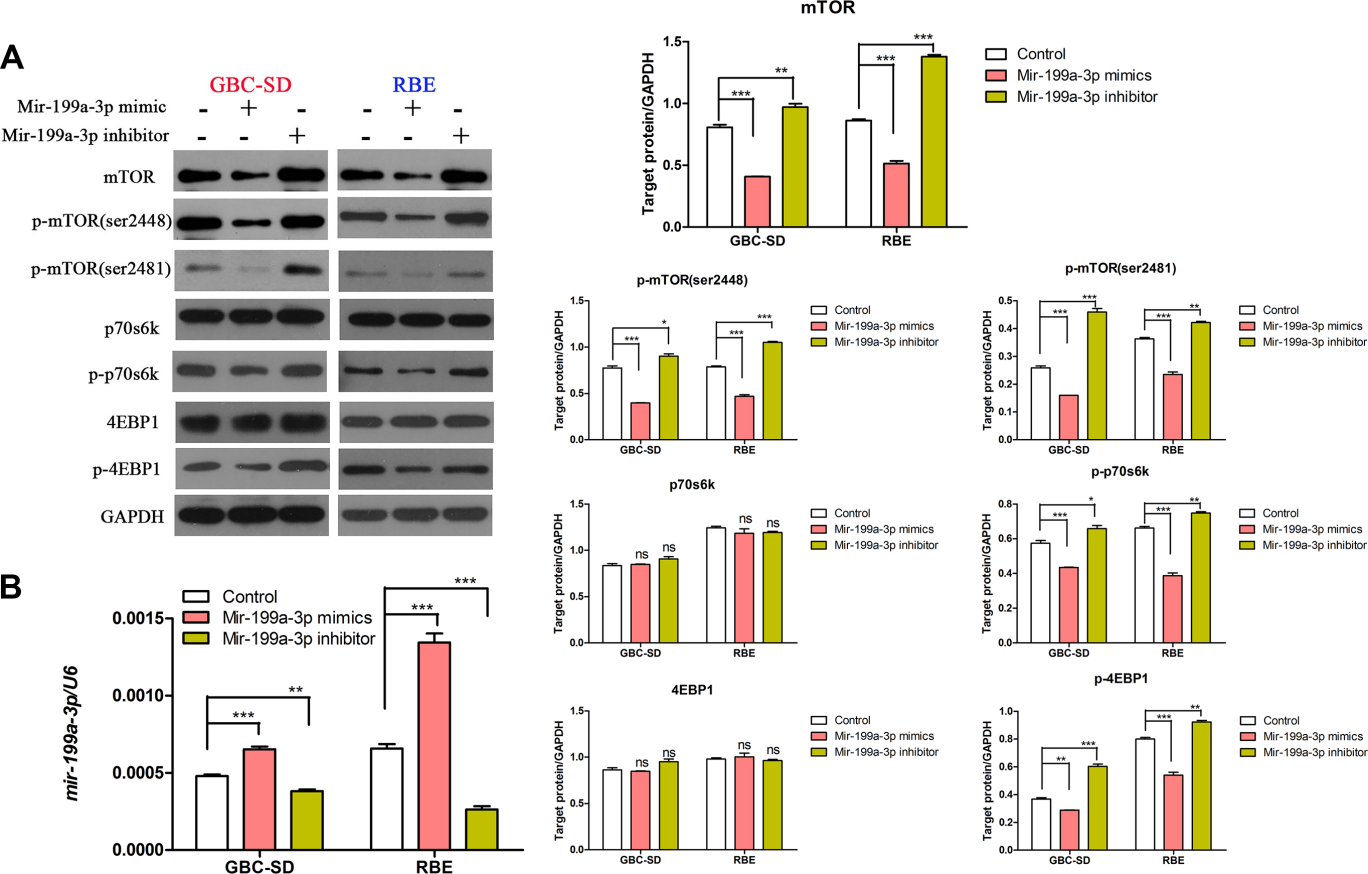
MiR-199a-3p could inhibit the mTOR signaling pathway (**A**) Expression of mTOR, p-mTOR (sites 2481 and 2448), p-4EBP1, p-p70s6k, 4EBP1 and P70s6K were detected by Western blotting in GBC-SD and RBE cell lines under different treatments with miR-199a-3p. (**B**) The efficiency of miR-199a-3p mimics and inhibitor were confirmed by qPCR analysis (**P < 0.01, ***P < 0.001).

To further investigate the mechanism of miR-199a-3p in regulating the sensitivity of cholangiocarcinoma cell lines to cisplatin, we used the target gene prediction site tool TargetScan (www.targetscan.org) to predict the downstream target gene. We found that positions 129–135 of the mTOR 3′-UTR had complementary pairing with miR-199a-3p (Figure 3A). Interestingly, cell line GBC-SD, which had lower expression of miR-199a-3p and higher expression of mTOR and p-mTOR (sites 2481 and 2448), while RBE had higher expression of miR-199a-3p, had lower expression of mTOR and p-mTOR (Figure 3B) (**P < 0.01, ***P < 0.001). To further confirm mTOR was the target gene of miR-199a-3p, we used Western blotting to detect the expression of mTOR signaling pathway proteins under the influence of miR-199a-3p mimics and inhibitor in GBC-SD and RBE cell lines. Compared to the negative controls, miR-199a-3p mimics led to the downregulation of total mTOR and p-mTOR, which consequently reduced the phosphorylation activation of mTOR downstream proteins 4EBP1 and p70s6k. Meanwhile, miR-199a-3p inhibitor caused the opposite result (Figure 4A) (**P < 0.01, ***P < 0.001). The efficiency of miR-199a-3p mimics and inhibitor was confirmed by PCR analysis (Figure 4B) (**P < 0.01, ***P < 0.001).

### MiR-199a-3p enhances the sensitivity of cholangiocarcinoma cell lines to cisplatin via suppression of mTOR

**Figure 5:**
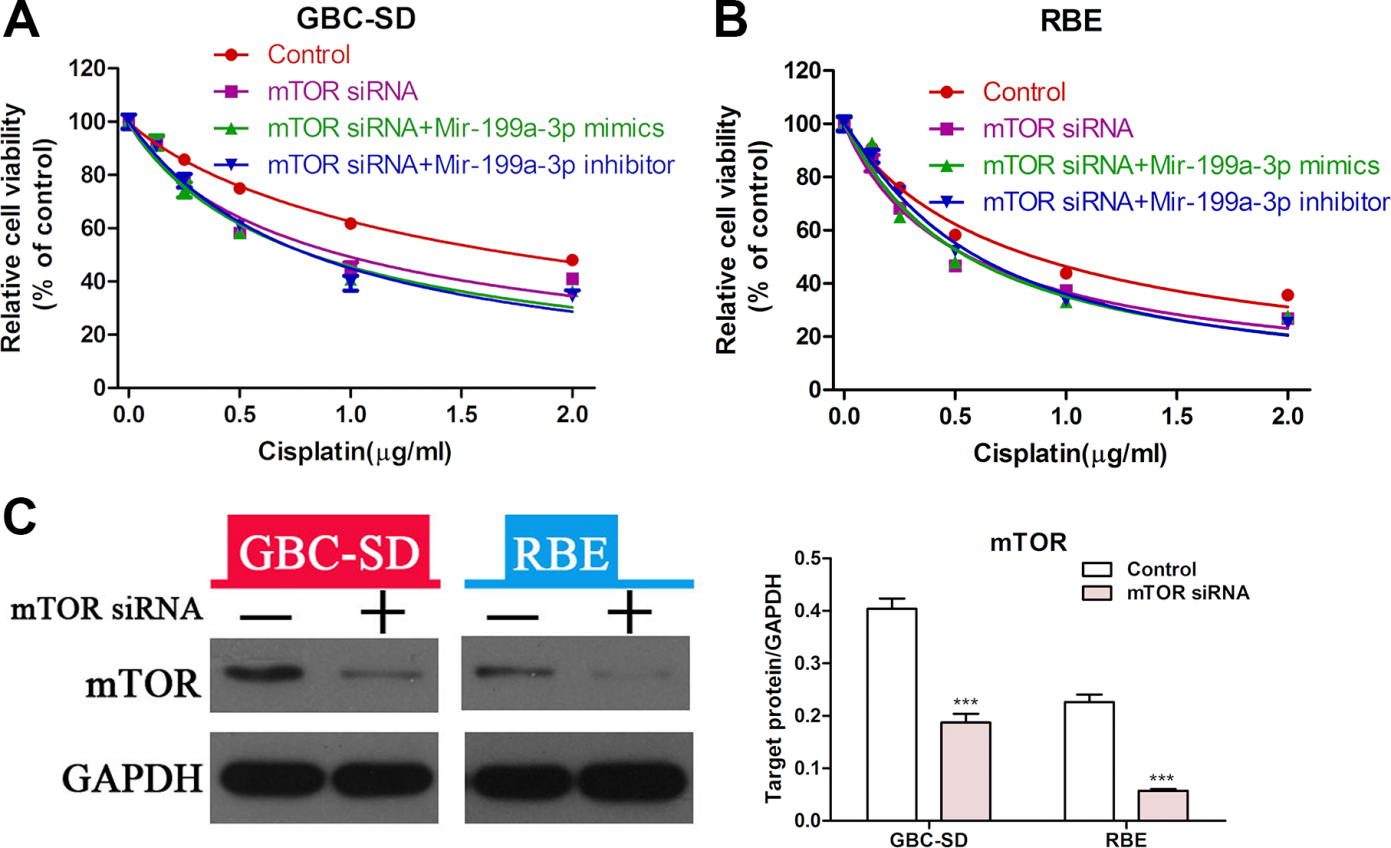
MiR-199a-3p enhanced cisplatin sensitivity of cholangiocarcinoma cells via the mTOR signal pathway (**A–B**) Cell viability of GBC-SD and RBE cell lines was detected by CCK-8 assay under different concentrations of cisplatin. Cells were treated by mTOR siRNA alone, negative control in combination with miR-199a-3p mimics or inhibitor. (**C**) Western blotting was used to confirm the efficiency of mTOR siRNA (***P < 0.001).

To further prove that miR-199a-3p could affect the sensitivity of cholangiocarcinoma to cisplatin by regulating the activity of the mTOR signaling pathway, we performed a rescue experiment. CCK-8 assay showed that suppression of mTOR led to greater sensitivity to cisplatin in GBC-SD and RBE cell lines, but the sensitivity of cholangiocarcinoma cells with mTOR knockdown to cisplatin was not changed when treated with miR-199a-3p mimics or inhibitor (Figure 5A–5B) (***P < 0.001). The efficiency of mTOR siRNA was confirmed by Western blot in both the GBC-SD and RBE cell lines (Figure 5C). These results indicated that the role of miR-199a-3p in regulating cisplatin sensitivity was mediated by the mTOR pathway.

### MDR1 was also involved in miR-199a-3p-mediated high-cisplatin-sensitivity in cholangiocarcinoma cells

**Figure 6:**
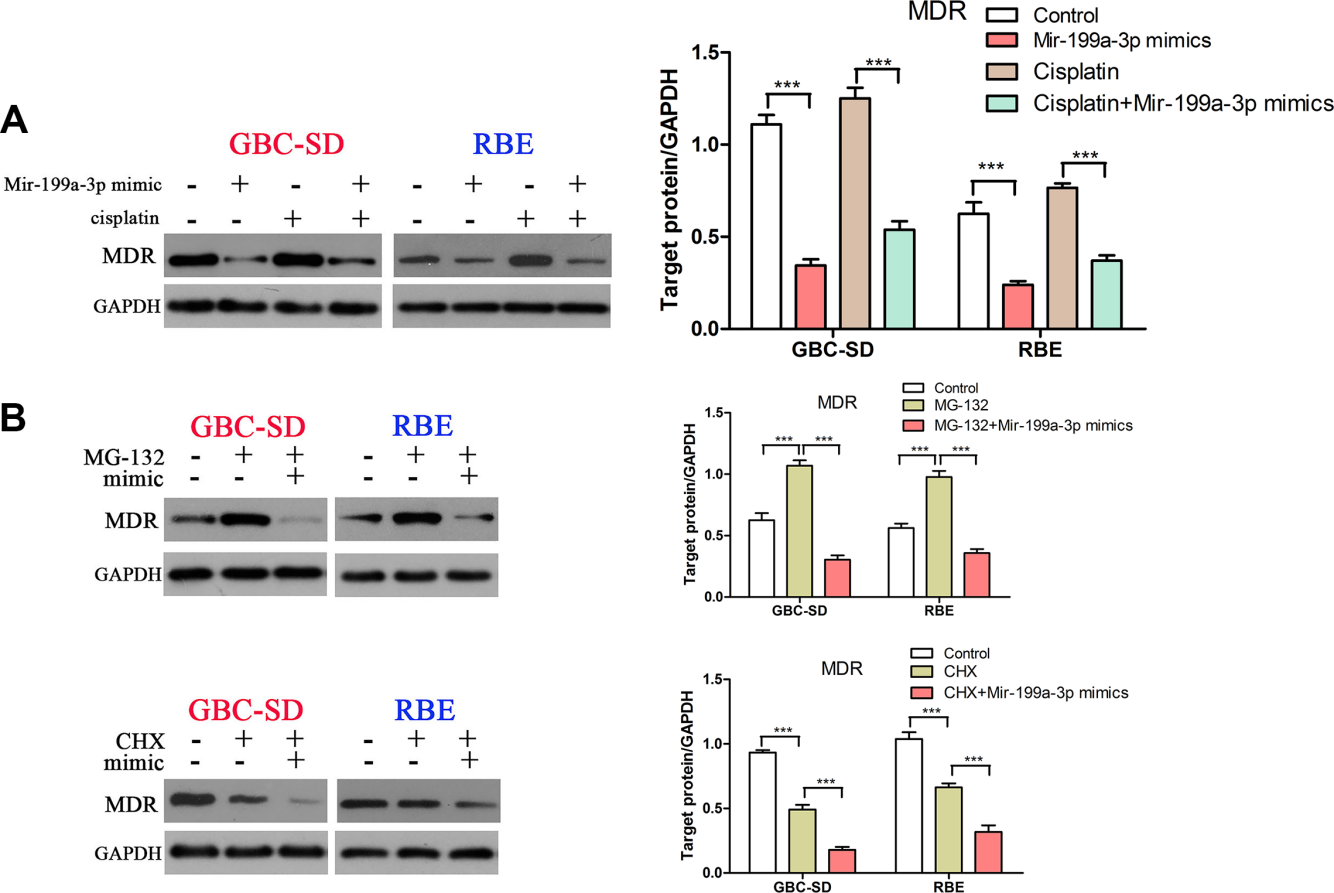
MiR-199a-3p inhibited cisplatin induced MDR1 expression (**A**) Protein level of MDR1 was detected in GBC-SD and RBE cell lines under treatment with miR-199a-3p mimics or cisplatin or both. The results were quantified using ImageJ software. (**B**) Protein expression of MDR1 was detected under treatment with MG-132 or miR-199a-3p mimics or both (upper panel), and the same with CHX (lower panel). The results were quantified using ImageJ (***P < 0.001).

Our previous study demonstrated that an mTOR inhibitor could increase the sensitivity of gallbladder carcinoma cells to 5-FU by downregulating expression of MDR1 [8]. Thus, we hypothesized that MDR1 may take part in miR-199a-3p-mediated high-cisplatin-sensitivity. As expected, we found that miR-199a-3p mimics could decrease cisplatin-induced MDR1 expression in GBC-SD and RBE cell lines (Figure 6A) (***P < 0.001). To further study the mechanism of miR-199a-3p in suppressing expression of MDR1, we used MG132 (a drug that can inhibit the ubiquitin degradation of MDR1) and CHX (a drug that can inhibit the synthesis of MDR1). We found that MG132 increased the protein level of MDR1 in GBC-SD and RBE cell lines, whilst using both MG132 and miR-199a-3p mimics led to lower expression of MDR1 compared to negative controls or the MG132 alone group. We also found that CHX decreased the protein level of MDR1 in GBC-SD and RBE cell lines, and using both CHX and miR-199a-3p mimics also led to lower expression of MDR1 compared to negative controls or the CHX alone group (Figure 6B) (***P < 0.001). These results demonstrated that miR-199a-3p regulated cisplatin sensitivity in cholangiocarcinoma cells by affecting the synthesis and degradation of MDR1.

## DISCUSSION

Cholangiocarcinoma is a highly malignant cancer with a poor prognosis. In clinically, gemcitabine plus cisplatin was associated with a significant survival advantage compared with gemcitabine alone in patients with locally advanced or metastatic cholangiocarcinoma cancer, suggesting that gemcitabine plus cisplatin is the standard regimen for advanced cholangiocarcinoma cancer [6, 23–24]. However, response to the combination chemotherapy in cholangiocarcinoma patients is limited, and the 5-year survival remains low [25]. Thus, we need to search for an effective therapeutic molecular that improve survival of cholangiocarcinoma patients.

MicoRNAs have been reported as playing a critical role in the regulation of key genes implicated in tumor proliferation, migration, and drug resistance [26]. Recent studies have reported that miR-199a-3p plays an important role in suppressing cell proliferation and migration in various tumors, such as prostate cancer [27], colorectal cancer [28], breast cancer [29], glioma [30], osteosarcoma [31] and pancreatic ductal adenocarcinoma [32]. Further studies have demonstrated that miR-199a-3p is a prognosis and diagnosis biomarker in hepatocellular carcinoma [33], gastric cancer [34] and colorectal cancer [35]. In addition, Fornari *et al*. reported that miR-199a-3p can regulate mTOR and c-Met to influence the doxorubicin sensitivity of human hepatocellular carcinoma cells [11]. However, the function of miR-199a-3p in cholangiocarcinoma was unknown. In this study, we focused on the role of miR-199a-3p in drug sensitivity of cholangiocarcinoma cells. Our results, for the first time, prove that miR-199a-3p can enhance cisplatin sensitivity in cholangiocarcinoma cell lines by regulating the mTOR signal pathway and MDR1.

MTOR, a ubiquitously expressed serine/threonine (Ser/Thr) kinase, is reported to play a crucial role in cancer progression by regulating cell proliferation, metabolism, drug resistance and other biological processes [36–38]. Inhibitors of mTOR, such as miRNAs, can inhibit tumor cell growth in many cancers by blocking the AKT/mTOR signaling pathway [39–41, 37]. Previous studies indicated that miR-199a-3p is an inhibitor of mTOR, and miR-199a-3p plays a role as a tumor suppressor by targeting mTOR in several cancers [18–20]. However, no study has reported the relationship between miR-199a-3p and mTOR in cholangiocarcinoma. Results from our study imply that miR-199a-3p plays a critical role as an inhibitor of mTOR in cholangiocarcinoma. Overexpression of miR-199a-3p led to a lower level of protein expression of mTOR and p-mTOR by binding the mTOR gene 3′-UTR and then regulating the phosphorylation status of 4E-BP1 and p70S6K. Above all, we demonstrated that miR-199a-3p could enhance the cisplatin sensitivity of cholangiocarcinoma by inhibiting the mTOR pathway.

ATP-binding cassette (ABC) transporter ABCB1 (P-glycoprotein/MDR1), a member of the ABC transporter family, has been investigated intensely for its association with drug resistance [42]. In our previous study, we demonstrated that MDR1 was regulated by the mTOR signaling pathway, and inhibition of the mTOR signaling pathway potently sensitized gallbladder cancer cells to 5-FU *in vitro* by suppressing the expression of 5-FU-induced MDR1 [10]; thus, we considered whether MDR1 was also involved in miR-199a-3p-mediated cisplatin-high-sensitivity in cholangiocarcinoma cells. Our results showed that the miR-199a-3p could reduce cisplatin-induced MDR1 expression, leading to high cisplatin sensitivity in cholangiocarcinoma cells. To further study the mechanism of miR-199a-3p in regulating MDR1 expression, we used two specific drugs to treat the cholangiocarcinoma cell lines. One was MG132, which can inhibit the ubiquitin degradation of MDR1 [43], the other was CHX, which can inhibit the synthesis of MDR1 [44]. As expected, the results showed that miR-199a-3p increased the cisplatin sensitivity of cholangiocarcinoma cell lines both by decreasing the synthesis of MDR1 and increasing the degradation of MDR1.

In conclusion, our study demonstrates that miR-199a-3p can increase the cisplatin sensitivity of cholangiocarcinoma cell lines by inhibiting the mTOR signaling pathway and MDR1 expression. This discovery may give novel insight into overcoming multidrug resistance and improving the prognosis of cholangiocarcinoma patients. However, further clinical and basic trials are needed to uncover the mechanisms underlying multidrug resistance in cholangiocarcinoma.

## MATERIALS AND METHODS

### Cell culture

Two human cholangiocarcinoma cell lines, RBE and GBC-SD, were purchased from the American Type Culture Collection (ATCC; Manassas, VA, USA) and cultivated according to the protocols described by ATCC. Cells were cultured in RPMI-1640 medium (Gibco, Grand Island, NY, USA) supplemented with 10% fetal bovine serum and maintained at 37°C in a humidified incubator under 5% CO_2_.

### Chemicals

MG132 and cycloheximide (CHX) were purchased from Sigma-Aldrich (St Louis, MO, USA). Cisplatin was purchased from Selleck (Houston, TX, USA).

### Cell viability assays

To detect relative cell viability, RBE and GBC-SD cell lines were seeded into 96-well microplates at a density of 5,000 cells per well. After 24 h for cell attachment, the culture medium was replaced with complete medium containing different concentrations of cisplatin (μg/ml) for 48 h. The cell counting kit-8 (CCK-8) assay (Dojindo, Kumamoto, Japan) was then performed according to the manufacturer's instructions. Briefly, 10 μL of CCK-8 working solution per 100 μL of medium was added into the microplates and the cells were incubated for 3 h. The OD 450 nm value was determined using a MRX II microplate reader (Dynex, Chantilly, VA, USA).

### EdU staining

RBE or GBC-SD cells were treated with miR-199a-3p mimics, inhibitor or negative control for 48 h. Then, under treatment with 1 μg/ml cisplatin for 48 h, EdU staining was performed by using the Click-iT EdU Imaging Kit (Invitrogen, Carlsbad, CA, USA) following the manufacturer's instructions. Three randomly selected fields of view per slide were selected under a fluorescence microscope (Olympus, Tokyo, Japan) and the number of proliferative cells (EdU positive) was counted.

### RNA interference

MiR-199a-3p mimics, inhibitors and negative control were synthesized by GenePharma (GenePharma Co. Ltd, China). SiRNAs specifically targeting mTOR were synthesized by Shanghai GeneChem Co. Ltd. (Shanghai, China). Transfection was conducted using lipofectamine 2000 (Invitrogen) according to the manufacturer's instructions. The knockdown efficiency of mTOR siRNA was determined by Western blot analysis, and the efficiency of miR-199a-3p mimics and inhibitor were confirmed by PCR analysis.

### Apoptosis analysis

RBE and GBC-SD cells were treated with miR-199a-3p mimics, inhibitor or negative control for 48 h, following which the culture medium was replaced with complete medium containing different concentrations of cisplatin (μg/ml) for 48 h. After this, cells were trypsinized and washed three times with prechilled phosphate-buffered saline (PBS) and resuspended in 100 μL PBS. Apoptotic RBE and GBC-SD cells were measured with APC-conjugated annexin-V and a propidium iodide (PI) kit according to the manufacturer's instructions (Dojindo, Kumamoto, Japan), and analyzed using a flow cytometer and FlowJo software.

### RT-PCR analysis

Total RNA was extracted from RBE and GBC-SD cell lines using TRIzol reagent (Invitrogen) following the manufacturer's protocol. First-strand cDNA was generated using a Reverse Transcription System Kit (Promega Corporation, Madison WI). Primers for miR-199a-3p were purchased from Takara (Dalian, China). Expression of miR-199a-3p was measured by qPCR with an ABI7500 Fast System (Applied Biosystems, CA) and SYBR green dye (Takara). U6 was used as an internal control, and the 2^−ΔΔCt^ method was used for relative quantification. All reactions were performed in triplicate.

mir-199a-3p inhibitor (human): 5′-UAACCAAUGUGCAGACUACUGU-3′mir-199a-3p mimics (human):Sense: 5′-UUCUCCGAACGUGUC ACGUTT-3′Antisense: 5′-ACGUGACACGUUCGG AGAATT-3′ Negative control: Sense: 5′-UUCUCCGA ACGUGUCACGUTT-3′Antisense: 5′-ACGUGACACG UUCGGAGAATT-3′

### Western blot analysis

Western blot analysis was performed as described previously [8]. The following antibodies were used: anti-mTOR (1:400 dilution; Cell Signaling, Natick, MA, USA), anti-p-mTOR (2481 and 2448) (1:1000; Cell Signaling), anti-p-4EBP1 (1:1000; Cell Signaling), anti-p-p70s6k (1:1000; Cell Signaling), anti-4EBP1 (1:1000; Abcam, Cambridge, UK), anti-P70s6K (1:1000; Abcam), anti-MDR1 (1:1000; Abcam), anti-GAPDH (1:2000; Abcam), and anti-HRP (1:2000; Cell Signaling).

### Statistical analysis

Three independent experiments were performed for each study. Comparisons among datasets were performed using one-way analysis of variance (ANOVA) tests or *t*-tests. *P < 0.05, **P < 0.01 and ***P < 0.001 were considered statistically significant.
